# Comparative Effectiveness of Semaglutide, Liraglutide, Orlistat, and Phentermine for Weight Loss in Obese Individuals: A Systematic Review

**DOI:** 10.7759/cureus.80321

**Published:** 2025-03-10

**Authors:** Jay P Patel, Daksh Hardaswani, Jaykumar Patel, Faizanali Saiyed, Rushita J Goswami, Taskin I Saiyed, Harshkumar Patel, Trishul H Amin

**Affiliations:** 1 Internal Medicine, Chirayu Medical College and Hospital, Bhopal, IND; 2 Internal Medicine, Jawaharlal Nehru Medical College, Wardha, IND; 3 Internal Medicine, Smt. Nathiba Hargovandas Lakhmichand (NHL) Municipal Medical College, Ahmedabad, IND; 4 Internal Medicine, Odessa National Medical University, Odessa, UKR; 5 Geriatrics, Bhavsinhji General Hospital, Porbandar, IND; 6 Otolaryngology, Smt. Nathiba Hargovandas Lakhmichand (NHL) Municipal Medical College, Ahmedabad, IND; 7 Internal Medicine, Gujarat Medical Education and Research Society (GMERS) Medical College and Hospital, Vadnagar, IND; 8 Internal Medicine, AMA School of Medicine, Makati, PHL

**Keywords:** comparative study, liraglutide or saxenda, medical weight loss, obesity, orlistat, phentermine, phentermine-topiramate, semaglutide, weight loss, weight-loss intervention

## Abstract

Obesity, a multifaceted and chronic condition characterized by excessive fat accumulation, poses significant risks to overall health and is associated with various metabolic and cardiovascular complications. This literature review evaluates and compares the effectiveness of four pharmacological agents semaglutide, liraglutide, orlistat, phentermine, and emerging agents like setmelanotide, amycretin, retatrutide, cagrilintide, and cotadutide in managing weight loss among obese. A detailed analysis was conducted on their mechanisms of action, dosing regimens, efficacy in weight loss, safety profiles, and their impact on obesity-related comorbidities. Although all agents presented distinct benefits, side effects such as gastrointestinal discomfort with orlistat and GLP-1 receptor agonists, and potential dependency with phentermine, necessitate tailored treatment approaches. This review highlights the importance of integrating pharmacotherapy with lifestyle interventions to achieve sustainable weight management and identifies areas for future research to optimize therapeutic outcomes for individuals with obesity.

## Introduction and background

Obesity is recognized as a multifaceted and long-term condition marked by the abnormal accumulation of body fat, which can negatively impact overall health [1]. Its development is influenced by a mix of genetic predispositions, environmental exposures, behavioral patterns, and metabolic factors [1]. This disorder is not uniform; instead, it manifests in various forms with unique biological and clinical characteristics. Additionally, it is frequently linked to several biomarkers that play a role in its progression and associated health complications [1]. Obesity occurs when there is an imbalance between the calories consumed and the energy expended, leading to an excessive buildup of fat in the body [2]. It is commonly evaluated using the body mass index (BMI), which is calculated by dividing a person’s weight in kilograms by the square of their height in meters [2]. Excessive weight gain has been escalating rapidly worldwide, with over one-third of the global population now classified as either overweight or obese [3]. This trend is evident across all age groups and genders, with a more pronounced impact observed among females. Additionally, obesity rates are often linked to the socioeconomic status of nations. As of 2015, an estimated 1.9 billion adults were classified as overweight, including approximately 609 million who were obese, collectively accounting for about 39% of the global population [3]. Recent global statistics reveal that over 1 billion individuals are affected by obesity, including approximately 880 million adults and 159 million children and adolescents as of 2022 [4]. Over the past few decades, obesity rates have surged significantly. Among women, the prevalence rose from 8.8% in 1990 to 18.5% in 2022, while in men, it nearly tripled from 4.8% to 14.0% during the same timeframe [4]. Similarly, childhood obesity has seen a steep increase, with rates for individuals aged 5-19 years climbing from 4% in 1975 to almost 20% by 2022. These trends underscore the escalating global challenge of obesity across all age groups [4].

Obesity can be classified using various methods, including body mass index (BMI), abdominal girth (AG), and body fat percentage (BF%). Each of these approaches has its own advantages and limitations. BMI is widely used to determine weight status due to its simplicity and low cost, as it only requires self-reported height and weight. In contrast, body fat percentage (BF%) can be assessed using several techniques, some of which involve advanced equipment and skilled technicians [5]. For instance, dual-energy X-ray absorptiometry (DXA), hydrostatic weighing, and air displacement plethysmography (such as the BodPod; Concord, CA: COSMED USA, Inc.) are highly accurate but require specialized resources. Alternatively, BF% can be measured through bioelectrical impedance analysis (BIA), which uses electrical currents to differentiate tissue types within the body [5]. Weight classifications based on BMI are as follows: individuals with a BMI over 25 kg/m² are categorized as overweight, while those exceeding 30 kg/m² are classified as obese. A BMI between 18.5 and 25 kg/m² is considered normal, whereas a BMI below 18.5 kg/m² indicates underweight status [5]. According to WHO guidelines, individuals with a BMI below 18.5 kg/m² are classified as underweight, while those within the 18.5-24.9 range fall under the normal weight category. A BMI of 25-29.9 is considered overweight, whereas obesity is further categorized into the following three classes based on severity: class I obesity (BMI: 30-34.9 kg/m²), class II obesity (BMI: 35-39.9 kg/m²), and class III obesity (BMI: ≥40 kg/m²). These classifications are widely utilized in clinical and research settings to assess obesity-related health risks and guide medical interventions [5]. A waist-to-hip ratio above 0.90 for men and 0.85 for women is widely recognized as indicative of an increased risk for obesity-related complications. Consequently, this metric is valuable not only for identifying obesity but also for predicting adverse health outcomes associated with abnormal fat distribution [6]. Obesity can result from a variety of mechanisms [7]. It has been attributed to an imbalance where energy intake significantly exceeds the energy expended by the body. This imbalance causes fat cells to enlarge abnormally, disrupting the nutrient signaling pathways that contribute to obesity [7]. However, recent research suggests that the source and quality of nutrients in a diet are more critical than their quantity for effective weight management and disease prevention [7]. Additionally, genetic predisposition plays a significant role in influencing an individual’s susceptibility to weight gain. Factors such as the accumulation of lipid metabolites, inflammatory processes, and dysfunction in hypothalamic neurons may also contribute to obesity, potentially explaining the biological mechanisms that favor the maintenance of excessive body fat [7]. Obesity is influenced by multiple factors, including dietary habits, energy balance, family history, lifestyle choices, gut microbiota, genetic predisposition, and epigenetic modifications [7]. Genetic mutations in leptin (LEP), leptin receptor (LEPR), proopiomelanocortin (POMC), melanocortin 4 receptor (MC4R), and fat mass and obesity-associated gene (FTO) play a pivotal role in obesity. These alterations interfere with appetite control, metabolic balance, and energy regulation, contributing to excessive fat accumulation and an elevated risk of obesity. Deciphering these genetic mechanisms is crucial for advancing precision medicine and developing targeted obesity interventions [2].

Recent research underscores the significance of small integral membrane protein 1 (SMIM1) - a gene encoding a small membrane protein - in regulating energy metabolism and body weight [8]. The study demonstrates that a deficiency in SMIM1 correlates with a marked reduction in energy expenditure, thereby increasing susceptibility to weight gain and obesity. Functionally, SMIM1 is integral to metabolic processes that sustain energy balance, likely by modulating pathways involved in energy utilization and lipid oxidation [8]. In scenarios where SMIM1 is either absent or its function is compromised, these critical metabolic pathways are less efficient, leading to diminished energy consumption and an accumulation of adipose tissue. Ultimately, these findings position SMIM1 as a crucial regulator of energy homeostasis, suggesting that its deficiency may predispose individuals to obesity and identifying it as a potential target for novel therapeutic interventions [8].

ABCA1 gene polymorphisms influence metabolic dysfunction in obesity by affecting high-density lipoprotein-very-low-density lipoprotein (HDL-VLDL) metabolism, insulin-glucose regulation, and inflammatory pathways. These variations may contribute to the development of metabolic syndrome and related complications [9]. The study revealed that certain variants are associated with decreased expression of ABCA1 in adipose tissue, which in turn impairs cholesterol efflux and disrupts normal lipid metabolism [9]. This impairment leads to the accumulation of lipids in fat cells, thereby contributing to higher body mass indices and an elevated risk of metabolic syndrome. Overall, these findings underscore the vital role of genetic factors in energy and lipid homeostasis and suggest that targeting metabolic genes like ABCA1 could offer promising new approaches for managing obesity and its related complications [9].

Inflammation and adipose tissue buildup in obesity involve several cytokines and immune cells. Pro-inflammatory cytokines such as tumor necrosis factor-alpha (TNF-α) and interleukin-6 (IL-6) are frequently elevated, leading to chronic low-grade inflammation and reduced insulin sensitivity [10]. Adipose tissue macrophages (ATMs) are also key players, as they shift to a pro-inflammatory state in obesity. This transition exacerbates inflammation and disrupts adipocyte function, ultimately impairing adipose tissue balance and contributing to broader metabolic disorders [10]. Obesity is linked to a wide range of complications, including cardiovascular conditions, type 2 diabetes, dyslipidemia, obstructive sleep apnea, and obesity hypoventilation syndrome [11]. It is also associated with osteoarthritis, liver disorders, polycystic ovary syndrome (PCOS), infertility, and mental health challenges such as depression, anxiety, and low self-esteem [11].

Behavioral interventions that encourage healthy eating and increased physical activity are recommended as the primary treatment for weight management [12]. However, these methods often fail to achieve and maintain significant weight loss (greater than 10%) due to adaptive mechanisms, such as reduced energy expenditure and heightened appetite, which act to prevent starvation. When lifestyle modifications alone are insufficient, pharmacological treatments may be introduced to support weight loss, with drug selection tailored to the patient’s existing comorbid conditions [12]. For individuals with severe obesity, bariatric surgery is an option, particularly for those with a BMI over 40 kg/m² or over 35 kg/m² accompanied by at least one comorbidity. Over time, pharmacological approaches to weight management have significantly advanced [12]. Phentermine, topiramate, orlistat, lorcaserin, naltrexone, liraglutide, and semaglutide (Wegovy) are among the drugs used for weight management, though semaglutide (Ozempic) has not yet been formally approved for weight loss [12].

This article aimed to provide an analysis of the effects of orlistat, phentermine, and semaglutide on weight loss, focusing on their mechanisms, impact on the body, and potential short- and long-term side effects. This article focuses on analyzing and comparing the efficacy of semaglutide, liraglutide, orlistat, and phentermine in promoting weight loss among individuals with obesity, based on a comprehensive review of existing studies. Furthermore, it examines their potential impact on managing obesity-related comorbidities. The review will evaluate the effectiveness of semaglutide, liraglutide, orlistat, and phentermine in managing obesity, focusing on weight loss outcomes, safety profiles, and mechanisms of action. It will also consider patient-specific factors, adherence, and cost implications while identifying gaps in current research and future directions for optimizing obesity treatment. Despite advancements in pharmacotherapy, lifestyle interventions including dietary modifications, physical activity, and behavioral counseling remain the foundation of effective and sustainable obesity management, emphasizing the need for a holistic, patient-centered approach to long-term weight control.

## Review

Methodology

This systematic review was designed to evaluate and compare the effectiveness of semaglutide, liraglutide, orlistat, and phentermine in managing obesity by following Preferred Reporting Items for Systematic Reviews and Meta-Analyses (PRISMA) guidelines [13]. The analysis systematically examined the mechanisms of action, dosing regimens, weight loss efficacy, safety profiles, and the impact of these pharmacological agents on obesity-related comorbidities. A thorough search of peer-reviewed journals and clinical trials was conducted using academic databases such as PubMed, Google Scholar, and Scopus. The search strategy employed key terms including "semaglutide efficacy in obesity management," "liraglutide for weight loss," "orlistat mechanism of action and effectiveness," "phentermine safety and efficacy in weight management," and "comparative analysis of obesity pharmacotherapies," with Boolean operators (AND, OR) applied to refine the results and achieve a thorough exploration of relevant studies. This review relied solely on publicly accessible data from previously published studies, without direct interaction with human participants, adhering to ethical standards. Proper citation and attribution were maintained for all referenced materials.

Inclusion and Exclusion Criteria

Articles published in peer-reviewed journals, studies involving adult participants with a BMI of ≥27 kg/m² or ≥30 kg/m², trials assessing weight loss outcomes, metabolic improvements, and safety profiles, and both clinical trials and review articles published between 2000 and 2024 were included in this study. Studies focusing on pediatric or adolescent populations and articles unrelated to the pharmacological agents under review were excluded from this study.

Data extraction, synthesis, and framework

The key details were systematically extracted from each study, including the research design (e.g., randomized controlled trials, retrospective studies, narrative reviews), sample size, participant demographics, treatment protocols (dose, frequency, and duration), outcomes such as weight loss percentage, metabolic improvements, and safety profiles, as well as the statistical significance of findings (e.g., p-values and confidence intervals). Data synthesis involved summarizing the results of each pharmacological agent, with quantitative outcomes like mean weight loss and percentage improvements organized into tables for clarity. Qualitative findings were analyzed narratively to provide a broader context in obesity management. The review employed a thematic framework, organizing findings into categories such as the mechanisms of action of each drug, dosing and administration guidelines, weight loss efficacy, safety profiles (both common and rare adverse effects), and the impact on obesity-related conditions, including diabetes and cardiovascular risks.

Search strategy

The search strategy incorporated Medical Subject Headings (MeSH) terms and relevant keywords, including “semaglutide,” “liraglutide,” “orlistat,” “phentermine,” “weight loss,” “anti-obesity treatment,” and “obesity management.” Boolean operators (AND, OR) were used to refine the results and exclude non-relevant studies. The table below summarizes the original search string and corresponding data (Table 1).

**Table 1:** The table summarized the original search string and corresponding data.

Database	Search string	Boolean operators	Filters applied	Year	Results retrieved
PubMed	((((((semaglutide) OR (liraglutide)) OR (orlistat)) OR (phentermine)) AND (weight loss)) AND (obesity)) OR (anti-obesity treatment)	AND, OR	Clinical trial, meta-analysis, randomized controlled trial	2000-2024	1317

Two independent reviewers screened the articles in a two-step process as follows: initially by title and abstract, followed by a full-text review of potentially eligible studies. Discrepancies between reviewers were resolved through discussion, and if consensus was not reached, a third reviewer provided arbitration.

Results

Extraction of Data

**Figure 1:**
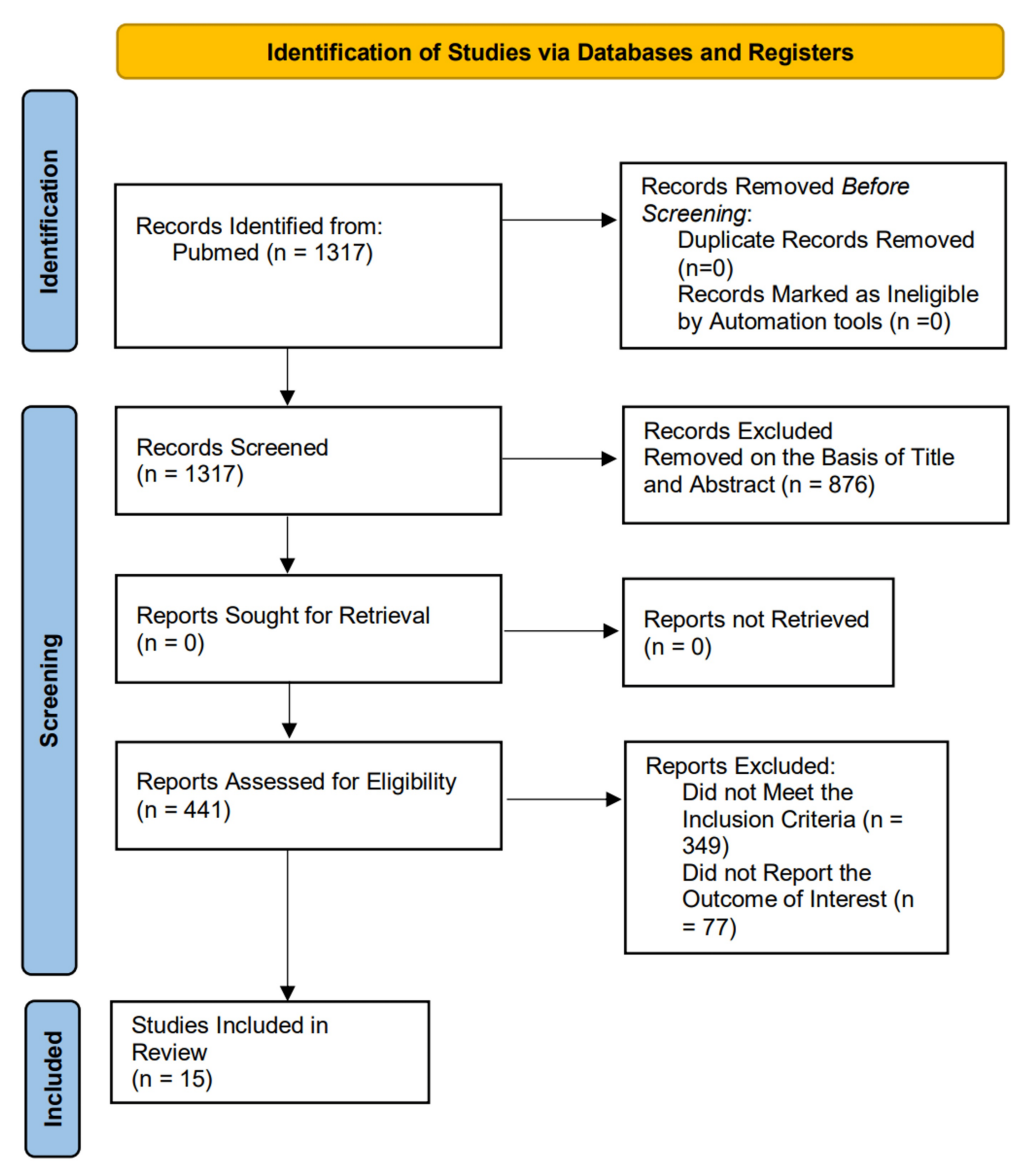
The study selection process is provided following the PRISMA flowchart. PRISMA: Preferred Reporting Items for Systematic Reviews and Meta-Analyses

A comprehensive search was conducted across PubMed, yielding a total of 1,317 records. After removing duplicates and ineligible records through automation tools (n=0), the remaining studies were screened by title and abstract. Following this step, 876 records were excluded due to irrelevance to the study objectives. A total of 441 full-text articles were assessed for eligibility. Of these, 349 studies were excluded for not meeting the criteria, while 77 did not report the relevant outcome of interest. Ultimately, 15 studies met the inclusion criteria and were incorporated into the final study. The study selection process is provided following the PRISMA flowchart (Figure 1).

Discussion

Obesity results from the interaction of genetic, environmental, and lifestyle factors [2]. The condition is largely driven by an imbalance between energy intake and expenditure, where the consumption of excess calories through food and beverages exceeds the energy burned through physical activity and metabolic processes [2]. The surplus energy is accumulated as fat, leading to an increase in body weight. Genetic factors also play a significant role in obesity, as some individuals may be predisposed to the condition due to variations in genes that regulate appetite, metabolism, and fat storage. Obesity is influenced by multiple genes, each having a small effect, with studies identifying genes involved in appetite regulation, fat metabolism, and energy balance, such as those related to leptin and its receptor [2].

Endocrine and metabolic factors are critical in the development of obesity [2]. One key mechanism is leptin resistance, a condition where the brain fails to respond to leptin, a hormone produced by fat cells that signals satiety. This resistance results in increased food consumption and continued weight gain [2]. Additionally, insulin resistance, another common metabolic issue associated with obesity, impairs the body's ability to respond to insulin, leading to elevated blood glucose levels that promote fat accumulation and contribute to inflammation. Adipose tissue in obese individuals also secretes dysregulated adipokines, which are hormones and cytokines that influence metabolic processes [2]. In obesity, levels of adiponectin decrease while pro-inflammatory cytokines like TNF-α and IL-6 increase, exacerbating insulin resistance and promoting chronic inflammation [2].

Obesity, particularly when associated with excess visceral fat, is characterized by chronic low-grade inflammation. This inflammation is primarily driven by an overproduction of inflammatory cytokines from adipose tissue, such as TNF-α, IL-6, and C-reactive protein (CRP) [14]. These inflammatory markers contribute to the development of metabolic disturbances, including insulin resistance and atherosclerosis, and increase the risk of other obesity-related comorbidities. Moreover, the gut microbiota composition in obese individuals differs from that of lean individuals, with an imbalance known as dysbiosis [2]. This condition can increase intestinal permeability, allowing endotoxins like lipopolysaccharides to enter the bloodstream, which in turn triggers systemic inflammation and contributes to metabolic dysfunction [2].

**Figure 2:**
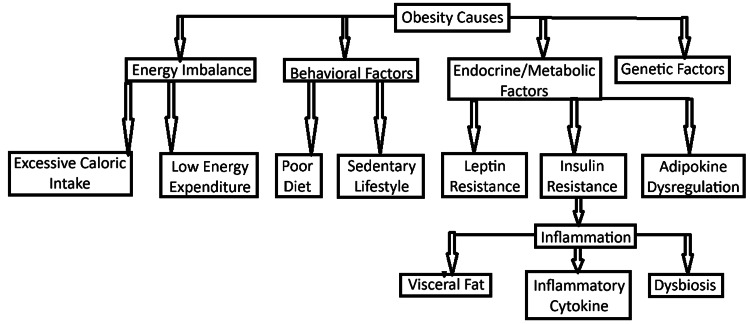
A flowchart of factors contributing to obesity. The flow chart highlights the interplay of genetic predisposition, environmental influences, behavioral patterns, hormonal imbalances, and metabolic dysfunctions, all of which interact to disrupt energy balance and promote excessive fat accumulation. The image is created by the author (Jay P Patel) of this study.

Behavioral factors also significantly impact the development and progression of obesity [2]. Poor dietary choices, such as the consumption of high-calorie and high-fat foods, coupled with sedentary lifestyles characterized by low physical activity, are primary contributors to obesity [2]. These behavioral factors, when combined with genetic predisposition, interact to disrupt the body’s ability to regulate weight, further fueling the obesity epidemic [2]. A summarized flowchart of factors contributing to obesity highlights the interplay of genetic predisposition, environmental influences, behavioral patterns, hormonal imbalances, and metabolic dysfunctions, all of which interact to disrupt energy balance and promote excessive fat accumulation (Figure 2).

Pharmacotherapy serves as an adjunct to lifestyle interventions, especially for individuals who struggle to achieve weight loss through lifestyle changes alone [15]. Surgical options are considered for individuals with severe obesity or when other treatments have not been successful. Combining these treatment modalities often yields the best outcomes [15]. Clinical practice guidelines emphasize the importance of a multidisciplinary approach tailored to the individual's needs, incorporating nutrition, physical activity, psychological support, pharmacotherapy, and surgical options when appropriate [15]. Several pharmacotherapies have been developed to aid weight loss, each varying in efficacy and mechanism of action. The article primarily focuses on evaluating the effectiveness of semaglutide (branded as Ozempic and Wegovy), liraglutide (Saxenda), orlistat, and phentermine.

Semaglutide

Mechanism of Action (MOA)

**Figure 3:**
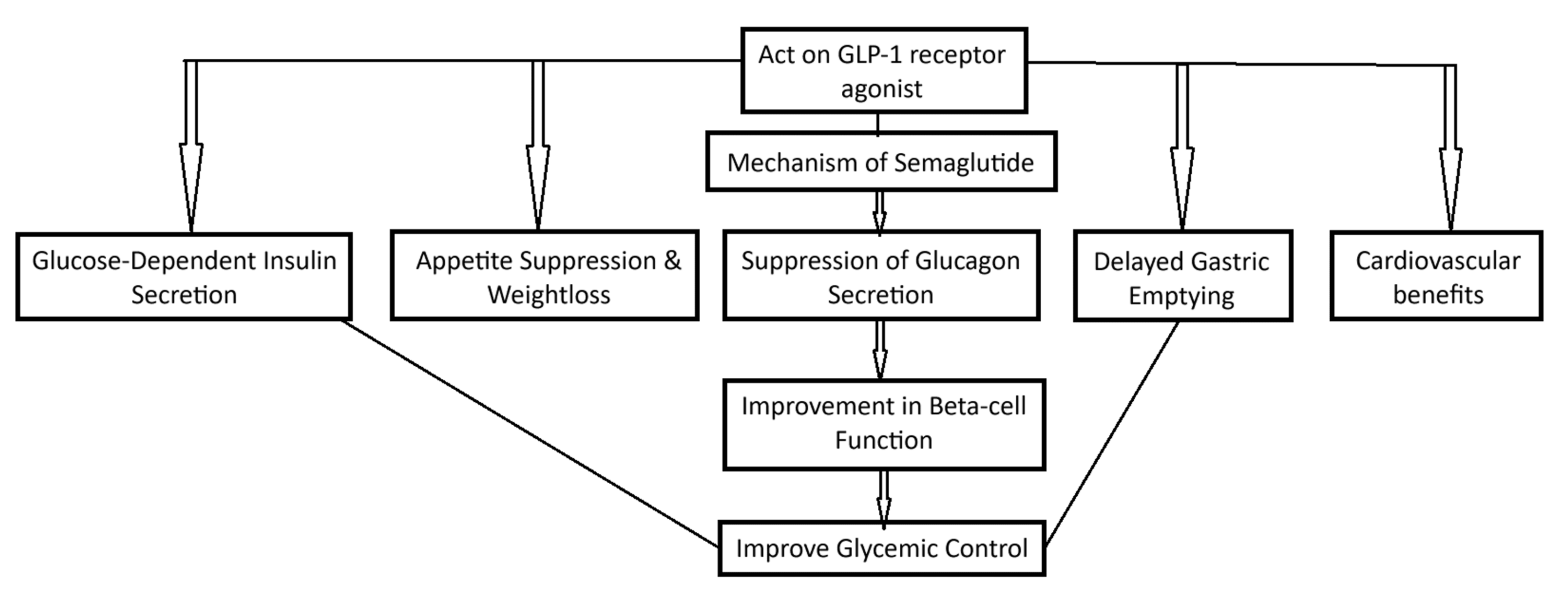
A flowchart of semaglutide's mechanism of action. The image is created by the author (Jay P Patel) of this study.

Semaglutide, a glucagon-like peptide-1 receptor agonist (GLP-1 RA), replicates the activity of the natural hormone GLP-1 to effectively regulate blood glucose levels and body weight through various mechanisms [16]. It enhances glucose-dependent insulin secretion by binding to GLP-1 receptors on pancreatic beta cells, which helps manage blood sugar levels while reducing the risk of hypoglycemia [16]. Additionally, semaglutide suppresses glucagon secretion from pancreatic alpha cells, thereby lowering hepatic glucose production and stabilizing blood sugar. By slowing gastric emptying, it reduces postprandial glucose spikes and ensures a more controlled nutrient absorption. Its action on GLP-1 receptors in the hypothalamus helps decrease appetite and caloric intake, significantly contributing to weight loss in patients [16]. Semaglutide also supports the preservation and function of pancreatic beta cells, potentially delaying the progression of type 2 diabetes. Furthermore, beyond its glycemic control benefits, semaglutide has demonstrated cardiovascular protective effects, including a reduction in major adverse cardiovascular events, making it a valuable therapeutic option for managing diabetes and associated comorbidities [16]. A summarized flowchart of semaglutide's mechanism of action (Figure 3).

Dose

Semaglutide is given for weight management at a higher dose [17]. The dosing regimen begins with an initial dose of 0.25 mg administered subcutaneously once a week for four weeks. The dose is then gradually increased to 0.5 mg weekly for another four weeks, followed by subsequent increases to 1.0 mg, and 1.7 mg, and ultimately reaching the maintenance dose of 2.4 mg per week after a 16-week dose escalation period [17]. This gradual titration helps improve tolerability and minimizes common side effects such as nausea, vomiting, and diarrhea. Semaglutide is indicated for adults with a body mass index (BMI) of 30 kg/m² or higher, classified as obese, or those with a BMI of 27 kg/m² or greater (overweight) accompanied by at least one weight-related comorbidity, such as hypertension, type 2 diabetes, or dyslipidemia [17].

Side Effects

The severity of semaglutide’s side effects increases with the dosage, with gastrointestinal issues being the most frequent and dose-dependent [17]. At lower doses, such as 0.25 mg or 0.5 mg weekly, common side effects include mild nausea, occasional vomiting, diarrhea, reduced appetite, mild headaches, and fatigue [17]. With moderate doses like 1.0 mg or 1.7 mg weekly, gastrointestinal symptoms such as nausea, more frequent vomiting, abdominal discomfort, dizziness, and constipation become more noticeable. At the highest dose of 2.4 mg weekly, severe gastrointestinal effects, including persistent nausea, significant vomiting, diarrhea, and abdominal pain, are common and may lead to dehydration or the need to discontinue treatment [17]. Higher doses are also associated with rare but serious complications such as pancreatitis, gallstones, and allergic reactions, as well as cardiovascular effects like increased heart rate and injection site reactions. Managing these side effects often involves gradual dose escalation, staying well-hydrated, and consuming smaller, low-fat meals. Regular monitoring by a healthcare provider is crucial to identify and address any severe or rare adverse effects promptly [17].

The study by Wilding et al. was a randomized, double-blind, placebo-controlled trial that assessed the efficacy and safety of once-weekly semaglutide in adults with overweight or obesity [17]. A total of 1961 participants were randomized in the trial, with 1,306 assigned to the semaglutide group and 655 to the placebo group. Approximately 74% of the participants were women, reflecting a predominance of female representation in the study population [17]. The trial included participants with a body mass index (BMI) of ≥30 kg/m² or ≥27 kg/m² with at least one weight-related comorbidity, excluding type 2 diabetes. Over 68 weeks, participants received semaglutide at a dose of 2.4 mg or a placebo, alongside lifestyle interventions such as diet and exercise [17]. The findings revealed a significant reduction in body weight among those receiving semaglutide, with an average weight loss of 14.9% compared to 2.4% in the placebo group, with all comparisons yielding p-values of less than 0.001. A higher proportion of participants in the semaglutide group achieved weight loss of ≥5%, ≥10%, and ≥15%. Secondary outcomes, including improvements in waist circumference, cardiometabolic risk factors (e.g., blood pressure, and lipid levels), and physical functioning, were also observed [17]. The study concluded that once weekly semaglutide, combined with lifestyle interventions, is highly effective for weight management, providing clinically meaningful and sustained weight loss and highlighting its potential as a therapeutic option for obesity management [17].

The SUSTAIN-6 trial conducted by Marso et al. primarily investigated the cardiovascular outcomes of semaglutide in 3,297 patients with type 2 diabetes assigned randomly who were at high cardiovascular risk [18]. This was a randomized, double-blind, placebo-controlled cardiovascular outcomes trial conducted over 104 weeks [18]. While the study's main focus was on cardiovascular safety, significant weight loss emerged as an important secondary benefit. Patients receiving semaglutide demonstrated considerable reductions in body weight compared to those given a placebo, with the weight loss being dose-dependent [18]. Participants treated with 0.5 mg and 1.0 mg weekly doses of semaglutide experienced notable improvements in both weight management and glycemic control. The study concluded that semaglutide not only reduces the risk of major adverse cardiovascular events (MACE) but also provides substantial weight loss benefits, reinforcing its potential as a comprehensive therapeutic option for managing weight, cardiovascular risk, and blood glucose in patients with type 2 diabetes [18]. The p-value for the weight loss outcome was reported as <0.001, indicating that the weight reduction observed in the semaglutide group was statistically significant compared to the placebo group [18].

The study by Ghusn et al. was a retrospective cohort study conducted at a weight management referral center. It included 175 adults with a BMI of 27 kg/m² or higher, classifying them as overweight or obese [19]. The participants had a mean age of 49.3 years, and 75.4% were women. Individuals with a history of bariatric surgery, those using other anti-obesity medications, or those with active malignant neoplasms were excluded. Participants received weekly subcutaneous injections of semaglutide, with doses titrated up to 1.7 mg or 2.4 mg, following established regimens used in clinical trials [19]. Data were collected from January 1, 2021, to March 15, 2022, with follow-up lasting up to six months to evaluate weight loss outcomes. The study reported statistically significant weight reductions, with a p-value of less than 0.001 [19]. On average, participants lost 5.9% of their body weight at three months and 10.9% at six months. Furthermore, 87.3% of participants lost at least 5% of their body weight, 54.9% achieved a reduction of 10% or more, and 23.5% experienced weight loss of 15% or greater. These findings are consistent with outcomes observed in randomized clinical trials, supporting semaglutide as an effective treatment for weight management in clinical practice [19].

The retrospective cohort study by Gasoyan et al. evaluated weight loss outcomes in 3,389 adults with a BMI of 30 kg/m² or greater who were treated with injectable semaglutide or liraglutide in clinical practice between July 1, 2015, and June 30, 2022 [20]. The findings demonstrated that, at one year, patients treated with semaglutide achieved a mean weight reduction of 5.1%, compared to 2.2% with liraglutide (p<0.001). Among patients treated for obesity, the average weight loss was 5.9%, while those treated for type 2 diabetes experienced a reduction of 3.2% (p<0.001) [20]. Continuous medication coverage significantly influenced outcomes, with a weight reduction of 5.5% observed in those with fewer than 90 days of cumulative gaps, compared to 2.8% for gaps between 90 and 275 days, and 1.8% for more than 275 days (p<0.001) [20]. Factors associated with achieving at least a 10% weight reduction included the use of semaglutide over liraglutide (aOR: 2.19; 95% CI: 1.77-2.72), treatment for obesity vs. type 2 diabetes (aOR: 2.46; 95% CI: 1.83-3.30), persistent medication coverage (aOR: 3.36; 95% CI: 2.52-4.54), higher dosage (aOR: 1.58; 95% CI: 1.11-2.25), and female sex (aOR: 1.57; 95% CI: 1.27-1.94). The study concluded that semaglutide is associated with greater weight loss compared to liraglutide and highlighted the importance of factors like treatment indication, medication coverage, and dosage in optimizing weight loss outcomes [20]. The key findings from four clinical and observational studies emphasize semaglutide's effectiveness and safety in managing weight and improving glycemic control. A summary of studies on semaglutide (Table 2). These studies, conducted across varied populations, revealed substantial weight reduction, enhanced cardiometabolic health, and decreased cardiovascular risks. The outcomes consistently showed superior results with semaglutide compared to placebo and liraglutide, highlighting its promise as a versatile treatment for obesity and associated health conditions.

**Table 2:** A summary of studies on semaglutide. MACE: major adverse cardiovascular events

Studies	Study type	Participants	Duration	Key findings
Wilding et al. [17]	Randomized, double-blind, placebo-controlled trial	1,961	68 weeks	Average weight loss: 14.9% (semaglutide) vs. 2.4% (placebo); p<0.001.
Marso et al. [18]	Randomized, double-blind, placebo-controlled cardiovascular outcomes trial	3,297	104 weeks	Weight loss dose-dependent; reduced risk of MACE; significant weight loss and glycemic control; p<0.001.
Ghusn et al. [19]	Retrospective cohort study	175	6 months	Weight loss: 5.9% at 3 months, 10.9% at 6 months; 87.3% lost ≥5%; 54.9% lost ≥10%; p<0.001.
Gasoyan et al. [20]	Retrospective cohort study	3,389	1 year	Weight loss: 5.1% (semaglutide) vs. 2.2% (liraglutide); p<0.001.

Liraglutide

Mechanism of Action (MOA)

Liraglutide is a glucagon-like peptide-1 receptor agonist (GLP-1 RA) that mimics the actions of the natural incretin hormone GLP-1 by binding to its receptors in the pancreas, brain, stomach, and cardiovascular system [16]. It enhances glucose-dependent insulin secretion by stimulating pancreatic beta cells when blood glucose levels are elevated, thereby minimizing the risk of hypoglycemia. Additionally, liraglutide inhibits glucagon secretion from pancreatic alpha cells, reducing hepatic glucose production and lowering fasting and postprandial blood glucose levels [16]. By slowing gastric emptying, it reduces postprandial glucose spikes and induces a sense of fullness. Liraglutide also acts on the hypothalamus to suppress appetite, leading to decreased caloric intake and weight loss [16]. Furthermore, it improves beta-cell function, reducing their workload and potentially slowing the progression of type 2 diabetes. Beyond glycemic control, liraglutide has cardioprotective effects, including a demonstrated reduction in cardiovascular events among patients with type 2 diabetes and established cardiovascular disease [16].

Dose

Liraglutide is an FDA-approved treatment option for weight management in adults who are obese or overweight and have associated comorbidities. The treatment starts with a daily subcutaneous injection of 0.6 mg for the first week [21]. To improve tolerability and minimize gastrointestinal side effects, the dosage is gradually increased by 0.6 mg each week over five weeks. By the fifth week, the recommended maintenance dose of 3.0 mg per day is typically reached [21]. The duration of therapy depends on the individual’s response, with progress evaluated after 16 weeks. If at least a 4% reduction in body weight is not achieved within this time frame, the medication is usually discontinued due to a lack of effectiveness [21]. Liraglutide is indicated for individuals with a body mass index (BMI) of 30 kg/m² or higher (obesity) or a BMI of 27 kg/m² or higher (overweight) who also have at least one related condition, such as type 2 diabetes, hypertension, or dyslipidemia [21].

Side Effects

Liraglutide, a GLP-1 receptor agonist, has several known side effects, with gastrointestinal issues being the most common and dose-dependent. Frequently reported symptoms include nausea, vomiting, diarrhea, constipation, and abdominal discomfort, especially during the initial stages of dose escalation. These effects generally diminish as the body adapts to the medication [21]. Additional side effects may include reduced appetite, fatigue, and headaches, which, while contributing to weight loss, can occasionally cause discomfort. Rare but serious adverse reactions include pancreatitis, gallbladder complications like gallstones (often linked to rapid weight loss), and hypersensitivity reactions. Mild injection site reactions, such as redness or swelling, may also occur [21]. Slight increases in heart rate have been observed as a cardiovascular side effect. Gradual dose increases and routine monitoring can help minimize these effects and enhance treatment tolerability [21].

The STEP 8 trial by Rubino et al. was a 68-week randomized, open-label study that evaluated the efficacy and safety of once-weekly semaglutide (2.4 mg) compared to daily liraglutide (3.0 mg) in adults without diabetes who were overweight or obese [22]. The study included 338 participants with a body mass index (BMI) of 30 kg/m² or higher, or 27 kg/m² or higher with at least one weight-related comorbidity, all of whom received lifestyle interventions alongside the treatments [22]. The primary outcome measured was the percentage change in body weight from baseline to week 68. Results demonstrated that semaglutide resulted in significantly greater weight loss compared to liraglutide, with a mean weight reduction of 15.8% vs. 6.4%, respectively, and a treatment difference of -9.4 percentage points (95% CI: -12.0 to -6.8; p<0.001) [22]. A higher percentage of participants achieved weight reductions of 5%, 10%, and 15% or more with semaglutide. Both treatments were generally well-tolerated, though gastrointestinal side effects were more common with semaglutide [22]. The findings of the trial concluded that semaglutide 2.4 mg administered weekly is significantly more effective than liraglutide 3.0 mg administered daily for weight loss in adults without diabetes who are overweight or obese [22].

The study conducted by Pi-Sunyer et al. was a 56-week randomized, double-blind, placebo-controlled trial designed to assess the efficacy and safety of liraglutide 3.0 mg for weight management in adults without type 2 diabetes [21]. The trial included 3,731 participants with a body mass index (BMI) of 30 kg/m² or higher, or 27 kg/m² or higher with conditions such as dyslipidemia or hypertension. Participants were randomly assigned in a 2:1 ratio to receive daily subcutaneous injections of liraglutide 3.0 mg (2,487 participants) or a placebo (1,244 participants) alongside lifestyle counseling [21]. At the end of the trial, those in the liraglutide group achieved an average weight loss of 8.4 kg, compared to 2.8 kg in the placebo group, with a significant treatment difference of -5.6 kg (95% CI: -6.0 to -5.1; p<0.001). Additionally, 63.2% of the liraglutide group achieved at least 5% weight loss vs. 27.1% in the placebo group, and 33.1% achieved over 10% weight loss compared to 10.6% in the placebo group (both p<0.001) [21]. Common side effects included mild-to-moderate nausea and diarrhea, which were typically temporary. The study concluded that liraglutide 3.0 mg, when combined with lifestyle changes, is an effective treatment for weight management in adults without diabetes [21].

The SCALE Maintenance study by Wadden et al. was a 56-week, randomized, double-blind, placebo-controlled trial that evaluated the effectiveness of liraglutide 3.0 mg administered daily in sustaining weight loss achieved through a low-calorie diet in overweight or obese adults without diabetes [23]. A total of 422 participants with a BMI of 30 kg/m² or higher, or 27 kg/m² or higher with related health conditions, were included, all of whom had lost at least 5% of their initial body weight during a pre-study low-calorie diet phase. Participants were randomly assigned to receive daily subcutaneous liraglutide or a placebo, alongside diet and exercise counseling [23]. Throughout the trial, the liraglutide group achieved an additional average weight loss of 6.2%, compared to 0.2% in the placebo group, with a difference of -6.1% (95% CI: -7.5 to -4.6; p<0.0001). Additionally, 81.4% of those treated with liraglutide sustained the initial ≥5% weight loss, compared to 48.9% in the placebo group, and 50.5% of liraglutide participants experienced an additional ≥5% weight loss, compared to 21.8% in the placebo group (both p<0.0001) [23]. The study also found that liraglutide improved cardiometabolic risk factors, while gastrointestinal side effects, though more common with liraglutide, were mild to moderate and temporary. The findings suggest that liraglutide 3.0 mg, combined with lifestyle interventions, is a beneficial option for maintaining and enhancing weight loss in overweight and obese individuals [23]. The table summarizes the studies on liraglutide (Table 3). The studies collectively highlight the efficacy of semaglutide and liraglutide for weight management in adults without diabetes who are overweight or obese. Semaglutide 2.4 mg weekly demonstrated superior weight loss compared to liraglutide 3.0 mg daily, while liraglutide was effective in both achieving and maintaining weight loss when combined with lifestyle interventions. Both treatments improved cardiometabolic risk factors, with gastrointestinal side effects being the most common but generally tolerable.

**Table 3:** A list and summary of the studies on liraglutide.

Studies	Study type	Participants	Duration	Key findings
Pi-Sunyer et al. [21]	Randomized, double-blind, placebo-controlled	3,731	56 weeks	Mean weight loss: 8.4 kg (liraglutide) vs. 2.8 kg (placebo); treatment difference: -5.6 kg (95% CI: -6.0 to -5.1; p<0.001).
Rubino et al. [22]	Randomized, open-label	338	68 weeks	Mean weight loss: 15.8% (semaglutide) vs. 6.4% (liraglutide); treatment difference: -9.4% (95% CI: -12.0 to -6.8; p<0.001).
Wadden et al. [23]	Randomized, double-bind, placebo-controlled	422	56 weeks	Additional weight loss: 6.2% (liraglutide) vs. 0.2% (placebo); difference: -6.1% (95% CI: -7.5 to -4.6; p<0.0001). Sustained ≥5% weight loss: 81.4% (liraglutide) vs. 48.9% (placebo). Additional ≥5% weight loss: 50.5% (liraglutide) vs. 21.8% (placebo).

Orlistat

Mechanism of Action (MOA)

Orlistat is a medication that inhibits gastrointestinal lipase, reducing the absorption of dietary fats in the digestive system. It achieves this by forming a covalent bond with the serine residue at the active sites of gastric and pancreatic lipases in the stomach and small intestine [24]. This action blocks these enzymes from breaking down triglycerides into free fatty acids and monoglycerides, which are the absorbable forms of fat. Consequently, about 30% of dietary fat is left undigested and eliminated in the feces, leading to a decrease in caloric intake and supporting weight loss. Additionally, orlistat contributes to improving lipid profiles, such as lowering LDL cholesterol [24]. With minimal systemic absorption, its effects are confined to the gastrointestinal tract, thereby minimizing the risk of systemic side effects [24].

Dose

Orlistat is a lipase inhibitor used to aid weight loss by reducing the absorption of dietary fats. The standard prescription dose is 120 mg, administered three times a day with each main meal that contains fat [24]. An over-the-counter version, marketed as Alli, is available in a 60 mg dose and is also taken with fat-containing meals. Orlistat is recommended to be taken either during a meal or within one hour after eating. If a meal is missed or contains no fat, the corresponding dose can be omitted. To minimize gastrointestinal side effects, such as oily stools, it's advisable to consume meals where no more than 30% of total calories come from fat [24]. Additionally, because orlistat can interfere with the absorption of fat-soluble vitamins (A, D, E, and K), it's recommended to take a multivitamin supplement at least two hours before or after taking orlistat, preferably at bedtime [24]. Orlistat is typically used as part of a comprehensive weight-loss plan that includes a reduced-calorie diet and regular physical activity. Its use should be monitored by a healthcare provider to ensure effectiveness and manage any potential side effects [24].

Side Effects

Orlistat may cause gastrointestinal side effects, including oily stools, flatulence, an urgent need to defecate, and increased bowel movements, particularly when consumed with meals high in fat [25]. Although these effects are typically mild and temporary, they can influence a patient’s willingness to continue treatment [25]. Additionally, orlistat can interfere with the absorption of fat-soluble vitamins (A, D, E, and K) and certain medications, such as cyclosporine and warfarin, necessitating appropriate supplementation and careful monitoring. The medication may also decrease the absorption of lipophilic drugs, potentially reducing their effectiveness [25]. Rare adverse events, such as severe liver damage and kidney injury associated with oxalate nephropathy, have been reported but occur infrequently [25].

The study conducted by Jain et al. assessed the effectiveness and safety of orlistat in managing obesity. This prospective, open-label study, conducted in India, involved 80 obese participants with a BMI of 30 kg/m² or higher, or 27 kg/m² or higher with associated conditions such as diabetes, hypertension, or dyslipidemia [26]. Participants were treated with orlistat (120 mg) three times daily for 24 weeks, alongside dietary adjustments and lifestyle counseling. The results revealed a significant reduction in weight, BMI, and waist circumference, with participants achieving an average weight loss of 5-10% of their initial body weight over the treatment period [26]. Additionally, improvements were observed in metabolic parameters, including fasting blood sugar and lipid profiles, with significant reductions in total cholesterol, triglycerides, and LDL levels. The reductions in weight, BMI, and waist circumference had a p-value of <0.001, improvements in lipid profiles had a p-value of <0.05, and fasting blood sugar improvements had a p-value of <0.01, all indicating statistically significant effects [26]. Gastrointestinal side effects, such as oily stools and flatulence, were mild and manageable, contributing to good adherence to treatment [26]. The study concluded that orlistat is a safe and effective option for weight management, providing additional metabolic benefits when combined with dietary and lifestyle modifications [26].

The study conducted by Krempf et al. was an 18-month multicenter, double-blind, randomized controlled trial aimed at evaluating the effectiveness of orlistat in promoting weight loss and maintaining it over the long term in obese individuals [27]. The trial included 696 participants aged 18-65 years with a body mass index (BMI) of 28 kg/m² or higher. Participants were randomly assigned to receive either 120 mg of orlistat or a placebo three times daily, alongside a mildly reduced-calorie diet maintained throughout the study duration [27]. Results showed that after 18 months, the orlistat group achieved a significantly greater average weight loss compared to the placebo group (-6.5% vs. -3.0% of initial body weight; p=0.0005). At 12 months, 32.9% of those receiving orlistat had lost at least 10% of their baseline body weight, compared to 24.5% in the placebo group (p=0.04) [27]. Furthermore, 28.1% of participants in the orlistat group maintained a ≥10% weight loss at 18 months, compared to 13.8% in the placebo group (p<0.0001). Orlistat also demonstrated metabolic benefits, including greater reductions in fasting blood glucose levels (-0.86 mmol/L vs. -0.29 mmol/L; p<0.05) and LDL cholesterol (-13.0% vs. -7.0%; p<0.001) [27]. The study concluded that orlistat, combined with a reduced-calorie diet, effectively promotes significant weight loss and long-term maintenance while improving metabolic risk factors associated with coronary heart disease [27].

The review by Sumithran and Proietto provides an in-depth analysis of the benefit-risk profile of orlistat in managing obesity [28]. This narrative review compiles findings from various clinical trials involving adults with obesity, defined as a BMI of 30 kg/m² or higher, or 27 kg/m² or higher with comorbid conditions. Orlistat, when combined with a reduced-calorie diet, was found to lead to a placebo-adjusted weight loss of approximately 3 kg over a year [28]. Additionally, it increased the likelihood of achieving a clinically significant weight reduction of at least 5% of initial body weight by around 20%. Beyond weight loss, orlistat showed benefits in improving systolic and diastolic blood pressure, lowering LDL cholesterol, enhancing glycemic control, and reducing the progression to diabetes in individuals with impaired glucose tolerance [28]. While orlistat generally has a favorable safety profile, gastrointestinal side effects such as oily stools and flatulence are common and can impact treatment adherence. Serious adverse events, including severe kidney and liver injury, are rare [28]. The authors conclude that orlistat provides modest but meaningful weight loss and metabolic improvements, though its gastrointestinal side effects may limit long-term use for some individuals [28].

The study by Sjöström et al. was a randomized, double-blind, placebo-controlled trial conducted across multiple centers to evaluate the efficacy of orlistat in promoting weight loss and preventing weight regain in obese patients [29]. The trial included 743 obese participants, aged 18-65 years, with a body mass index (BMI) ranging from 28 to 43 kg/m². Participants were randomly assigned to receive either 120 mg of orlistat or a placebo three times daily, in conjunction with a hypocaloric diet during the first year, followed by a weight maintenance diet in the second year. After one year, the orlistat group experienced a significantly greater mean weight loss compared to the placebo group (-10.2% vs. -6.1% of initial body weight; p<0.001) [29]. During the second year, participants who continued orlistat treatment regained less weight than those who switched to placebo (32% vs. 58% regained ≥50% of lost weight; p<0.001). Additionally, orlistat treatment was associated with significant improvements in obesity-related risk factors, including reductions in total cholesterol, low-density lipoprotein (LDL) cholesterol, and fasting glucose levels [29]. The study concluded that orlistat, combined with dietary intervention, is effective in achieving significant weight loss and reducing weight regain, along with improving obesity-related risk factors in obese patients [29]. The table below summarizes the studies on orlistat (Table 4). The studies on orlistat demonstrate its effectiveness in achieving significant weight loss, maintaining long-term weight reduction, and improving metabolic parameters such as lipid profiles, glycemic control, and blood pressure. Common gastrointestinal side effects are manageable, supporting its role as a safe and effective option for obesity management.

**Table 4:** A table summarizes the studies on orlistat.

Studies	Study type	Participants	Duration	Key findings
Jain et al. [26]	Prospective, open-label	80	24 weeks	Weight loss: 5-10% of initial body weight; significant improvements in BMI and waist circumference (p<0.001).
Krempf et al. [27]	Multicenter, double-blind, randomized controlled	696	18 months	Weight loss: -6.5% (orlistat) vs. -3.0% (placebo) after 18 months (p=0.0005).
Sumithran et al. [28]	Narrative review of clinical trials	_	_	Placebo-adjusted weight loss of ~3 kg/year; ~20% increased likelihood of ≥5% weight reduction.
Sjöström et al. [29]	Randomized, double-blind, placebo-controlled	743	2 years	Year 1: -10.2% (orlistat) vs. -6.1% (placebo) weight loss (p<0.001); year 2: lower weight regain with orlistat (32% vs. 58%; p<0.001).

Phentermine

Mechanism of Action (MOA)

Phentermine (PHEN) is a sympathomimetic amine that helps reduce appetite. It works by stimulating the release of norepinephrine and dopamine within the central nervous system, increasing their levels in the synaptic cleft [30]. This enhanced neurotransmitter activity activates the hypothalamus, the brain region responsible for controlling appetite, leading to a decrease in hunger and reduced food consumption [30]. Furthermore, phentermine’s influence on norepinephrine pathways also contributes to a rise in energy expenditure, supporting its role in weight management [30].

Dose

PHEN is commonly prescribed for weight loss at a dosage of 15-37.5 mg once daily, administered before breakfast or 1-2 hours after breakfast [31]. Phentermine is often combined with topiramate to aid in weight loss. The combination therapy of phentermine and topiramate was evaluated in two dosing regimens for weight management in overweight and obese adults [32]. The low-dose regimen consisted of phentermine 7.5 mg paired with topiramate 46 mg, taken once daily, while the high-dose regimen included phentermine 15 mg combined with topiramate 92 mg, also taken once daily. Both dosing strategies were implemented alongside lifestyle modifications to assess their effectiveness in promoting weight loss [32].

Side Effects

Common side effects of phentermine include an increased heart rate (tachycardia), higher blood pressure, palpitations, restlessness, difficulty sleeping, dizziness, dry mouth, diarrhea, and constipation [33]. In less common cases, individuals may experience feelings of euphoria, followed by fatigue or depression. Rarely, psychotic episodes or hallucinations can occur. Additionally, due to its stimulant nature, there is a risk of dependence and potential misuse [33].

The CONQUER trial study done by Gadde et al. was a randomized, double-blind, placebo-controlled, phase 3 study that evaluated the efficacy and safety of a phentermine-topiramate combination in 2,487 adults aged 18-70 years with a BMI of 27-45 kg/m² and at least two weight-related comorbidities such as hypertension, dyslipidemia, or diabetes. Participants received lifestyle counseling along with either a placebo, a low-dose combination (phentermine 7.5 mg/topiramate 46 mg daily), or a high-dose combination (phentermine 15 mg/topiramate 92 mg daily) [32]. After 56 weeks, mean weight loss was 1.4% in the placebo group, 8.1% in the low-dose group, and 10.2% in the high-dose group (p<0.0001 for both doses vs. placebo). Improvements in metabolic parameters, including blood pressure, triglycerides, and fasting glucose, were also observed, with higher remission rates of prediabetes and metabolic syndrome in the treatment groups [32]. The most common side effects were dry mouth, constipation, paraesthesia, and insomnia, occurring more frequently at higher doses but generally mild-to-moderate. The study demonstrated that the phentermine-topiramate combination is effective for weight loss and improving metabolic health, offering a valuable option for long-term obesity management [32].

The article by Smith et al. provides a comprehensive review of the pharmacology, efficacy, and safety of the phentermine/topiramate (PHEN/TPM) combination therapy for obesity management [34]. The authors analyzed data from three Phase 3 clinical trials - EQUIP, CONQUER, and SEQUEL - involving obese patients. These studies demonstrated that PHEN/TPM treatment resulted in statistically significant weight loss compared to placebo [34]. Specifically, after 56 weeks, weight reductions were 10.6% for the 15/92 mg dose, 8.4% for the 7.5/46 mg dose, and 5.1% for the 3.75/23 mg dose, with all comparisons yielding p-values less than 0.0001. The SEQUEL trial, a 52-week extension, indicated sustained weight loss over two years, with reductions of 9.3% and 10.5% from baseline for the 7.5/46 mg and 15/92 mg doses, respectively [34]. The review also addressed safety concerns, noting that common adverse effects included dry mouth, constipation, paraesthesia, insomnia, and dizziness, which were generally mild-to-moderate in severity. The authors concluded that PHEN/TPM is an effective and well-tolerated option for long-term obesity treatment [34].

The study published in 2016 by Thomas et al. investigated factors predicting weight loss success with phentermine treatment in obese individuals [35]. Conducted as a prospective cohort study, it included 35 participants with a BMI ranging from 30 to 40 kg/m², who participated in an eight-week weight loss program with daily administration of 15 mg of phentermine [35]. The findings revealed that participants with higher hunger levels and lower dietary restraint at baseline achieved greater weight loss, showing a statistically significant correlation (p<0.05). These results highlight that baseline appetite-related traits may be key predictors of weight loss outcomes during phentermine treatment [35].

The study by Kang et al. was a randomized, double-blind, placebo-controlled trial designed to evaluate the efficacy and safety of a newly developed diffuse-controlled release (DCR) formulation of phentermine in obese individuals [36]. The trial included 74 participants with controlled comorbid conditions such as diabetes, hypertension, or dyslipidemia. Participants were randomly assigned to receive either 30 mg of phentermine DCR once daily (n=37) or a placebo (n=37) for 12 weeks [36]. Results showed that the phentermine DCR group achieved a significant mean weight loss of 8.1±3.9 kg compared to 1.7±2.9 kg in the placebo group (p<0.001). Waist circumference reductions were also significantly greater in the phentermine group (p<0.001) [36]. Additionally, the phentermine group showed improvements in metabolic parameters, including lipid profiles and blood pressure, though specific p-values for these improvements were not reported. The most commonly reported side effects were dry mouth and insomnia, which were mild-to-moderate in severity [36]. The study concluded that phentermine DCR 30 mg daily is an effective and well-tolerated short-term treatment option for obesity, leading to significant weight loss and reductions in waist circumference [36]. The table below summarizes the studies on phentermine (Table 5). The studies highlight the efficacy and safety of phentermine-topiramate and phentermine formulations for obesity management. The combination therapy demonstrated significant weight loss and metabolic improvements across multiple trials, with sustained effects over two years. Phentermine alone showed promising short-term results, with baseline appetite traits influencing outcomes. Common side effects, such as dry mouth and insomnia, were generally mild, supporting the use of these treatments in appropriate patients.

**Table 5:** A list and summary of the studies on phentermine. PHEN: phentermine; TPM: topiramate

Studies	Study type	Participants	Duration	Key findings
Gadde et al. [32]	Randomized, double-blind, placebo-controlled, phase 3 trial	2,487	56 weeks	Placebo: 1.4% weight loss; low dose: 8.1% weight loss; high dose: 10.2% weight loss; p<0.0001 for both doses vs placebo.
Smith et al. [34]	Comprehensive review of 3 phase 3 trials: EQUIP, CONQUER, SEQUEL	EQUIP: 1,267; CONQUER: 2,487; SEQUEL: 676	EQUIP, CONQUER: 56 weeks; SEQUEL: 108 weeks	10.6% weight loss for (PHEN/TPM) 15/92 mg; 8.4% weight loss for 7.5/46 mg; 5.1% weight loss for 3.75/23 mg (p<0.0001); sustained weight loss in SEQUEL trial: 9.3% and 10.5% for 7.5/46 mg and 15/92 mg, respectively.
Thomas et al. [35]	Prospective cohort study	35	8 weeks	Participants with higher hunger levels and lower dietary restraint achieved greater weight loss (p<0.05).
Kang et al. [36]	Randomized, double-blind, placebo-controlled trial	74	12 weeks	Weight loss: 8.1±3.9 kg (phentermine) vs. 1.7±2.9 kg (placebo) (p<0.001); significant waist circumference reduction (p<0.001).

The following baseline characteristic data were extracted from each included article such as author, total participants, mean age, duration of the study, mean BMI, intervention, and outcome (Table 6). Tirzepatide, a dual GIP/GLP‑1 receptor agonist, has demonstrated significant efficacy in weight reduction. At a 15 mg dose, certain cohorts experienced weight losses of up to 22.5%. In patients with type 2 diabetes, weight reductions between 9.5% and 11.3% were observed over 40 weeks. Among individuals with obesity, the treatment has been shown to reduce initial body weight from 15% to 20%, with the extent of loss being dose-dependent [37].

**Table 6:** The baseline characteristics of the included studies. PHEN: phentermine; TPM: topiramate

Studies	Total participants	Mean age	Duration	Mean BMI	Intervention	Outcome
Wilding et al. [17]	1,961	46 years	68 weeks	38 kg/m²	Once-weekly subcutaneous semaglutide at a dose of 2.4 mg, combined with lifestyle intervention vs. placebo.	Semaglutide group weight reduction of 14.9%, compared to a 2.4% reduction in the placebo group; p<0.001.
Marso et al. [18]	3,297	64 years	104 weeks	32.2 kg/m²	Once-weekly semaglutide (0.5 mg or 1.0 mg) or placebo.	The mean body weight in the semaglutide group, as compared with the placebo group, was 2.9 kg lower in the group receiving 0.5 mg and 4.3 kg lower in the group receiving 1.0 mg; p<0.001.
Ghusn et al. [19]	175	49 years	6 months	41 kg/m²	Weekly 1.7 mg or 2.4 mg semaglutide subcutaneous injections.	Weight loss: 5.9% at 3 months, 10.9% at 6 months; p<0.001.
Gasoyan et al. [20]	3,389	50 years	1 year	40 kg/m²	Injectable forms of semaglutide or liraglutide.	Weight loss: 5.1% (semaglutide) vs. 2.2% (liraglutide); p<0.001.
Pi-Sunyer et al. [21]	3,731	46 years	56 weeks	38.5 kg/m²	Once-daily subcutaneous injections of liraglutide at a dose of 3.0 mg or placebo.	Liraglutide group achieved a mean weight loss of ~8.4% from baseline vs. ~2.8% in the placebo group.
Rubino et al. [22]	338	47 years	68 weeks	38 kg/m²	Weekly subcutaneous semaglutide 2.4 mg vs. daily liraglutide 3.0 mg.	Mean weight loss: 15.8% (semaglutide) vs. 6.4% (liraglutide); p<0.001.
Wadden et al. [23]	422	47 years	56 weeks	38 kg/m²	Once‑daily liraglutide 3.0 mg vs. placebo.	Liraglutide group achieved an additional mean weight loss of about 6.1% from randomization vs. approximately 4.2% in the placebo group.
Jain et al. [26]	80	40 years	24 weeks	35 kg/m²	Group 1 patients received orlistat 120 mg three times a day vs. group 2 patients received placebo three times a day.	Mean percentage weight reduction seen in orlistat-treated group was 5.63% compared to 2.3% in placebo-treated group.
Krempf et al. [27]	696	41 years	18 months	36.1 kg/m²	Orlistat 120 mg or placebo three times daily in conjunction with a mildly reduced-energy diet.	A significantly higher proportion of orlistat-treated patients, compared with placebo, achieved a ≥10% reduction in their initial body weight after 12 and 18 months; p<0.0001.
Sumithran et al. [28]	_	≥12 years	_	≥27 kg/m^2^	Orlistat treatment in conjunction with a hypocaloric diet compared with placebo.	Participants receiving orlistat compared with placebo lost >5% of starting weight.
Sjöström et al. [29]	743	44 years	2 years	36 kg/m²	Orlistat 120 mg (three times a day) or placebo in conjunction with the hypocaloric diet.	At 12 months, the orlistat group achieved a mean weight loss of approximately 10.2% of their initial body weight compared with about 6.3% in the placebo group. In year 2, patients who switched from placebo to orlistat lost an additional 0.9 kg on average, whereas those remaining on placebo regained 2.5 kg; p<0.001.
Gadde et al. [32]	2,487	51 years	56 weeks	36.2 kg/m²	Placebo or a combination of controlled-release phentermine plus topiramate administered as two active doses (initial low-dose {7.5 mg phentermine/46 mg topiramate} titrated to 7.5/46 mg, and a higher dose to 15/92).	Placebo: 1.4% weight loss; Low dose: 8.1% weight loss; High dose: 10.2% weight loss; p<0.0001.
Smith et al. [34]	EQUIP – 1,267, CONQUER- 2,487, SEQUEL- 676	EQUIP- 42 years, CONQUER- 51 years, SEQUEL- 52 years	EQUIP and CONQUER- 52 weeks, SEQUEL- 52 weeks extension study of CONQUER	EQUIP, CONQUER, and SEQUEL- 42, 36.6, and 36.1 kg/m², respectively	Across these trials, the 3 PHEN/TPM doses studied were 3.75/23 mg (low-dose), 7.5/46 mg (mid-dose), and 15/92 mg (high-dose).	10.6% weight loss for (PHEN/TPM) 15/92 mg; 8.4% weight loss for 7.5/46 mg; 5.1% weight loss for 3.75/23 mg (p<0.0001); sustained weight loss in SEQUEL trial: 9.3% and 10.5% for 7.5/46 mg and 15/92 mg, respectively.
Thomas et al. [35]	35	37 years	8 weeks	33.8 kg/m²	Participants were given an 8-week supply of 30 mg phentermine hydrochloride tablets and were instructed to take one tablet every morning at the same time.	Participants with higher hunger levels and lower dietary restraint achieved greater weight loss; p<0.05.
Kang et al. [36]	74	35 years	12 weeks	33 kg/m²	Phentermine diffuse controlled release 30 mg or placebo.	Phentermine diffuse controlled release group showed significant reductions in body weight (-8.1±3.9 vs. -1.7±2.9 kg, p<0.001) and waist circumference (7.2±0.5 vs. 2.1±0.6 cm, p<0.001).

Tuccinardi et al. 2019 performed a six‐month, randomized, placebo‐controlled, double‐blind trial to assess both the efficacy and safety of lorcaserin in an obese population. In this study, participants were allocated to receive either lorcaserin - a selective 5-HT_2_C receptor agonist that increases satiety by activating hypothalamic pro-opiomelanocortin neurons - or a placebo [38]. The results revealed that those treated with lorcaserin achieved an average weight loss of about 5% from their baseline body weight, a statistically significant improvement compared to the negligible change seen in the placebo group. Additionally, lorcaserin treatment was accompanied by improvements in several cardiometabolic risk factors, highlighting its potential utility in obesity management. The drug was generally well tolerated, with the most frequently reported adverse events being headache, dizziness, nausea, and dry mouth, all of which were predominantly mild-to-moderate in severity [38].

Emerging agents

Amycretin is emerging as a promising investigational agent in obesity pharmacotherapy. It is engineered to merge the pharmacological effects of both amylin and incretin receptor agonists, thereby leveraging the synergistic advantages of these hormonal pathways. By targeting mechanisms that regulate glycemic control and appetite simultaneously, amycretin aims to enhance satiety, reduce caloric intake, and ultimately facilitate weight loss. Although the clinical evidence remains preliminary, early data indicate that the drug may provide significant efficacy with a tolerable safety profile. Ongoing and future large-scale clinical trials will be critical to substantiate its long-term benefits and clarify its potential role in the management of obesity [37].

Setmelanotide has been assessed in phase 3, single-arm, open-label, multicenter trials involving individuals with severe obesity due to POMC or LEPR deficiency. Daily administration of setmelanotide in these studies resulted in notable reductions in hunger and significant weight loss. The most frequently reported adverse effects included injection site reactions, nausea, and vomiting, which were typically mild-to-moderate in severity. These findings emphasize setmelanotide’s potential as a targeted therapy for obesity linked to genetic defects in the leptin-melanocortin pathway. However, further long-term studies are needed to establish its sustained efficacy and safety in this population. Clinical trial data revealed that 80% of participants with POMC deficiency and 45% of those with LEPR deficiency experienced at least a 10% reduction in body weight over one year of treatment. Notably, some individuals achieved weight reductions exceeding 20%, with a few reaching 25% or more. These results further support setmelanotide’s efficacy as a precision therapy for patients with specific genetic forms of obesity [37].

Retatrutide, a novel triple receptor agonist, concurrently targets the GLP-1, GIP, and glucagon receptors to enhance energy expenditure and suppress appetite. In a phase 2 clinical trial involving 338 adults with obesity, the highest administered dose (12 mg) resulted in an average weight reduction of 24.2% over 48 weeks. The most frequently reported adverse events were gastrointestinal, including nausea, diarrhea, vomiting, and constipation. These effects were dose-dependent, generally mild-to-moderate in severity, and primarily observed during dose escalation. Notably, adverse event frequency was greater in the 8 mg and 12 mg dose groups, particularly among participants who initiated treatment at 4 mg rather than 2 mg. Refinement of the dose-escalation strategy in phase 3 trials may help mitigate these side effects and optimize tolerability [39-41].

Cagrilintide a long-acting amylin analogue, is currently in phase 2 clinical trials for weight management. In a randomized, double-blind, placebo-controlled, and active-controlled dose-finding trial, once-weekly administration of cagrilintide in adults with overweight or obesity led to dose-dependent weight loss. The most frequently reported adverse events were gastrointestinal-related, primarily nausea and vomiting, which were generally mild-to-moderate in severity and typically resolved within 48 hours. These preliminary findings indicate that cagrilintide holds potential as an effective therapeutic option for obesity management, though further validation through larger, long-term clinical studies is required [39-41].

Additionally, cotadutide, a dual GLP-1 and glucagon receptor agonist, is currently being evaluated in phase 2 clinical trials involving adults with type 2 diabetes and varying degrees of renal impairment. These studies have shown that cotadutide induces modest weight loss, with reductions averaging 5-10%, while also significantly enhancing glycemic control and metabolic parameters. The most frequently observed adverse effects are gastrointestinal-related, primarily nausea and vomiting, which generally diminish over time. Further investigation through larger, long-term trials is necessary to establish its efficacy and safety profile for broader clinical use [39-41].

Limitations

The article predominantly reviews studies conducted in controlled clinical environments, which may not accurately reflect real-world conditions or patient adherence. Variability in study designs, sample sizes, durations, and outcome measures complicates direct comparisons between the findings. Furthermore, the focus is largely on the efficacy of individual medications, with limited discussion on combination therapies that are becoming increasingly significant in obesity treatment. A notable limitation is the emphasis on short-term outcomes (typically 12-68 weeks), offering minimal insight into the long-term effectiveness, safety, and sustainability of weight loss. Additionally, the review provides limited analysis of the cost-effectiveness and affordability of these pharmacological treatments, which are critical factors for practical implementation and broader accessibility.

Future strategies

Long-term research is essential to assess the sustained effectiveness, safety, and tolerability of semaglutide, liraglutide, orlistat, and phentermine. Evaluating the prolonged metabolic and cardiovascular effects of these treatments will improve their practical application in clinical settings. Further, research on emerging agents such as amycretin, setmelanotide, retatrutide, cagrilintide, and cotadutide should be expanded, with a focus on their long-term benefits, potential synergies, and comparative efficacy against existing therapies. Future investigations should also emphasize real-world scenarios to understand how these medications function beyond controlled trials. This includes examining patient compliance, the integration of these treatments with lifestyle modifications, and their effectiveness across diverse populations with different socioeconomic and cultural contexts.

## Conclusions

Semaglutide demonstrates superior efficacy in weight loss, achieving the highest percentage of weight reduction and additional cardiometabolic benefits. Liraglutide is effective but less potent and requires daily administration. Phentermine offers short-term benefits but raises concerns about dependency and cardiovascular risks. Orlistat provides modest weight loss but has gastrointestinal side effects that may affect adherence. Hence, semaglutide is the preferred option for significant and sustained weight loss. Ultimately, the selection of a pharmacological treatment should be tailored to individual patient needs, factoring in obesity severity, existing comorbidities, and medication tolerability. Combining pharmacological therapies with lifestyle changes remains crucial for achieving and maintaining long-term weight management. Based on the reviewed studies, the average weight loss achieved with these pharmacological agents is approximately 15% for semaglutide, 8.4% for liraglutide, 8-10% for phentermine, and 5-10% for orlistat.
